# Is myopia accelerated in type 1 diabetes mellitus children? Analyses from the ocular parameters

**DOI:** 10.1186/s12886-023-02908-2

**Published:** 2023-04-11

**Authors:** Ying Xiao, Yu Qian, Chenhao Yang, Haidong Zou

**Affiliations:** 1 Department of Ophthalmology, Children’s Hospital of Fudan University, National Children’s Medical Center, 399 Wanyuan Road, Shanghai, 201102 China; 2 Department of Ophthalmology, Shanghai General Hospital (Shanghai First People’s Hospital), Shanghai Jiao Tong University School of Medicine, Shanghai, China; 3 Shanghai Key Laboratory of Fundus Diseases, Shanghai, China; 4 Shanghai Eye Diseases Prevention & Treatment Center, Shanghai Eye Hospital, Shanghai, China; 5 National Clinical Research Center for Eye Diseases, Shanghai, China; 6 Shanghai Engineering Center for Precise Diagnosis and Treatment of Eye Diseases, Shanghai, China

**Keywords:** T1DM children, Lens thickness, Lens power, Myopia

## Abstract

**Background:**

This study compares the ocular biometry with or without myopia in children with type 1 diabetes mellitus (T1DM) and healthy children in China to analyse the difference between myopia in T1DM and healthy children.

**Methods:**

A case-control study was conducted at the Children’s Hospital of Fudan University. The children were divided into four subgroups depending on myopia or non-myopia, T1DM or non-DM. The participants were evaluated for anterior chamber depth (ACD), lens thickness (LT), axial length (AL), average keratometry (K) and lens power (P). Furthermore, cycloplegic refraction was performed and the spherical equivalent (SE) was acquired.

**Results:**

One hundred and ten patients with T1DM and 102 healthy subjects were included in this study. In the age-sex adjusted analysis, the myopia T1DM subgroup showed thicker LT (p = 0.001), larger P (p = 0.003) and similar ACD, AL, K and SE (all p > 0.05) compared to the myopia control subgroup. Additionally, the myopia T1DM subgroup showed longer AL (p < 0.001) and similar ACD, LT, K and P (all p > 0.05) as the non-myopia T1DM subgroup. In the multivariate linear regression, for T1DM patients, eyes with longer AL, shallower ACD, and larger P were associated with a decrease in SE (p < 0.001, p = 0.01, and p < 0.001, respectively). Meanwhile, for healthy controls, eyes with longer AL and larger P were associated with a decrease in SE (all p < 0.001).

**Conclusions:**

The ACD and LT of myopia T1DM children remained unchanged compared to non-myopia T1DM children. This means that the lens in the former group could not lose power as compensation for AL growth, thus providing evidence for the acceleration of myopia in T1DM children.

## Introduction

To maintain the eye’s refractive status, its axial length (AL), corneal power, and lens power (P) need to be in balance. AL elongation has contributed to more than 70% of myopia progression, followed by P and corneal power [1]. However, since the cornea hardly changes in diameter and power after year 2 [2], the role of corneal power in myopia progression is limited. In this context, apart from AL, more attention should be paid to P.

In a study conducted on chickens’ eyes from age 10 to 90 days, AL was observed to increase while the cornea and the lens lost power. While the lens became larger and its curvature flattened, its equivalent index decreased. Subsequently, as a result of all these changes, the lens lost 30 diopters of power during eye growth in chickens. This means that refractions were maintained in the low hyperopic range [3]. In human beings, the lens is compacted inside the nucleus, accompanied by a slow rate of the addition of new fibres in the newly developed cortex after birth. As a result, not only does the lens start thinning in the first 10 years of life, but the gradient index power also decreases because of the nucleus compaction reflecting a more abrupt climbing gradient profile, contributing to the lens losing its internal power [2]. Therefore, in childhood, the lens compensates for AL elongation to maintain refractive status. However, myopia might develop once the lens reaches its power loss limit because of its internal structure [2]. The CLEERE Study Group found that myopia onset is characterised by an abrupt loss of compensatory lens changes that continue in emmetropes throughout the period of childhood AL elongation [4], which accelerates before the onset of myopia, while the highest spherical equivalent (SE) progression occurs during the year of onset [5]. Although myopic eyes have thinner lenses, lower P, and longer anterior segment lengths to compensate for longer AL [2, 6], they exhibit a lesser P reduction than non-myopes. This decreased ability for P loss, in turn, may be a result of not only internal structure limitations but also growing age and increasing AL [7].

Moreover, lens compensation for AL elongation is not an endless process—myopia occurs when AL increases to a certain extent. Under such circumstances, what would be the consequences if the lens suffered from some other condition, such as hyperglycaemia? In our previous ocular biometric study conducted on children with type 1 diabetes mellitus (T1DM), an increase in lens thickness (LT) was accompanied by a decrease in anterior chamber depth (ACD). At the same time, when SE was observed to remain unaffected compared to healthy controls, compensation by the lens refractive index was suspected [8]. Considering that one-half of P changes result from LT and lens curvature [2], a question that arises is whether the compensation by the lens refractive index is sufficient to address LT growth and AL elongation in myopia T1DM children. Furthermore, the progress of P in children with myopia and T1DM requires in-depth investigations. In view of these concerns, uncertainties arose regard to lens compensation ability and whether myopia is accelerated in children with T1DM. To address these gaps, this study was conducted to examine and further elucidate the differences between ocular biometry changes in T1DM myope and non-DM myope children.

## Methods

This was a hospital-based case-control study approved by the ethics committee of both Children’s Hospital of Fudan University in Shanghai (approval number: No. 01 (2018)) and Shanghai General Hospitals (approval number: 2016KY005). This study conformed to the guidelines proposed in the Helsinki Convention. It was a part of the Shanghai Children and Adolescent DM Eye study (SCADE). SCADE is a study aiming to investigate the ocular disorders of DM children since January 2018, and we arranged yearly follow-ups. New enrollment is around every January; the deadline here was 2021.

Patients with T1DM who had enrolled within the four years were included in this study. Meanwhile, healthy subjects were chosen from among the children who came to the clinic for routine vision examinations and were willing to participate in our research conducted in January 2018 and January 2019. Patients with other metabolic disorders (i.e., Prader-Willi syndrome) and those under myopia control measures (i.e., orthokeratology or atropine eye drops) were excluded from the sample. Eyes with a history of ocular trauma and diseases (i.e., corneal pathology, cataract, glaucoma, optic nerve atrophy, retinopathy and strabismus) were also excluded. Written informed consent and a medical history questionnaire were obtained from each participant’s parent.

The methodology followed in this study has already been published [8]. Notably, the same team performed the examinations in this study. The refractive error, as well as K1 and K2 keratometry, was measured by an autorefractor (ARK-1; Nidek, Tokyo, Japan), while the keratometry data was converted to average K, K = (K1 + K2)/2. The ACD, LT and AL were acquired by utilizing IOL Master (700; Carl Zeiss Meditec, Dublin, CA). Following this, the pupil was dilated with 1% cyclopentolate before performing subjective refraction. The refraction data were converted into the spherical equivalent (SE; SE = sphere power + 1/2 cylinder power). Furthermore, the refractive power of the lens (P) was calculated using the modified Bennette-Rabbetts formula [9, 10].

Eyes were classified into one of two groups based on their SE – the myopia group consisted of those with SE of at least − 0.50 D (inclusive), while the rest formed the non-myopia group. Subsequently, each group was divided into two subgroups – the T1DM group and the control group.

Statistical analysis of the data was performed using SPSS version 26.0. Mean values and standard deviations (SDs) were used for descriptive analyses. Furthermore, an independent T-test was conducted to compare the differences between the continuous variables in the subgroups, the Pearson chi-square test was employed for carrying out gender analyses between the subgroups, while univariate general linear models were used to compare the differences in ocular biometry between the subgroups, adjusting for age and sex. In addition, multivariate linear regression analyses were performed to explore the association between SE and ACD, LT, AL and P, after adjusting for the age and sex of the T1DM and non-DM groups. Considering the high correlation between both eyes, only the left eye was selected for statistical analysis. Plots of the mean and standard error of the ACD, LT, AL and P for different ages are in terms of both eyes. All data were approximately normally distributed, while the statistical significance was set at p < 0.05.

## Results

The study included 110 patients with T1DM and 102 healthy subjects. The mean ages of the myopia T1DM and myopia control subgroups were 13.04 ± 3.04 years and 10.31 ± 2.11 years, respectively. The difference between these two groups was found to be significant (p < 0.05). The mean age of the non-myopia T1DM and non-myopia control subgroups were 9.50 ± 2.85 years and 8.30 ± 1.90 years, respectively, with the difference between the two being significant (p < 0.05). Furthermore, there were 20 males and 30 females in the myopia T1DM group, while the myopia control group consisted of 43 males and 32 females – the difference between the two was found to be insignificant (p = 0.058). At the same time, there were 30 males and 30 females in the non-myopia T1DM group, while 14 males and 13 females comprised the non-myopia control group – indicating an insignificant difference between the two groups (p = 0.873).

Table 1 presents comparisons of the means and SDs obtained from the ocular biometry of the myopia T1DM and myopia control subgroups on the one hand and the non-myopia T1DM and non-myopia control subgroups on the other. The data presented below were adjusted for age and sex. The LT was significantly thicker in the myopia T1DM group than the myopia control group (p = 0.001), and the ACD of the myopia T1DM group was shallower than the myopia control group, but the difference was not significant (p > 0.05). Furthermore, the myopia T1DM group exhibited a significantly larger P than the myopia control group (p = 0.003). However, the non-myopia T1DM and non-myopia control subgroups showed no difference in the case of all parameters (ACD, LT, AL, K, P and SE, all p > 0.05).

**Table 1:** Ocular parameters comparations between subgroups of T1DM patients and healthy controls, Mean ± SD

	Myopia T1DM N = 50	Myopia control N = 75	p-value	Non-myopia T1DM N = 60	Non-myopia control N = 27	p-value
ACD, mm						
Unadjusted	3.45 ± 0.31	3.52 ± 0.30	0.266	3.32 ± 0.28	3.22 ± 0.37	0.194
Age-sex adjusted	3.43 ± 0.05	3.53 ± 0.04	0.103	3.30 ± 0.04	3.25 ± 0.06	0.412
LT, mm						
Unadjusted	3.46 ± 0.16	3.34 ± 0.15	< 0.001	3.50 ± 0.21	3.50 ± 0.16	0.979
Age-sex adjusted	3.46 ± 0.03	3.34 ± 0.02	0.001	3.51 ± 0.03	3.48 ± 0.04	0.547
AL, mm						
Unadjusted	24.72 ± 1.14	24.66 ± 1.04	0.763	23.08 ± 0.67	23.00 ± 1.04	0.694
Age-sex adjusted	24.48 ± 0.14	24.82 ± 0.11	0.091	23.03 ± 0.10	23.11 ± 0.15	0.686
K, D						
Unadjusted	43.22 ± 1.47	43.09 ± 1.59	0.639	43.02 ± 1.18	43.25 ± 1.42	0.460
Age-sex adjusted	43.17 ± 0.24	43.13 ± 0.20	0.893	43.02 ± 0.17	43.24 ± 0.26	0.486
P, D						
Unadjusted	22.53 ± 1.80	21.75 ± 1.65	0.018	23.88 ± 1.97	23.31 ± 2.63	0.309
Age-sex adjusted	22.72 ± 0.26	21.61 ± 0.22	0.003	23.95 ± 0.28	23.15 ± 0.44	0.133
SE, D						
Unadjusted	-3.22 ± 2.27	-2.24 ± 1.73	0.011	0.50 ± 0.70	0.63 ± 0.93	0.474
Age-sex adjusted	-2.72 ± 0.27	-2.57 ± 0.22	0.703	0.53 ± 0.10	0.58 ± 0.15	0.787

ACD: anterior chamber depth; LT: lens thickness; AL: axial length; K: average keratometry; P: lens power; SE: spherical equivalent; D: diopters; SD: standard deviation. p-value < 0.05 significant.

Table 2 compares the means and SDs obtained from the ocular biometry of the myopia T1DM and non-myopia T1DM subgroups, as well as the myopia control and non-myopia control subgroups. The data presented below were adjusted for age and sex. Between the two control subgroups, the myopia subgroup exhibited deeper ACD (p = 0.001), thinner LT (p = 0.001), longer AL (p < 0.001) and smaller P (p = 0.028). Furthermore, between the two T1DM subgroups, the myopia subgroup showed a longer AL (p < 0.001), but similar LT (p = 0.824), ACD (p = 0.579) and P (p = 0.110) values. Notably, K was stable for both pairs of subgroups (all p > 0.05).

**Table 2:** Ocular parameters comparations between subgroups of myopia subjects and non-myopia subjects, Mean ± SD

	Myopia T1DM N = 50	Non-myopia T1DM N = 60	p-value	Myopia control N = 75	Non-myopia control N = 27	p-value
ACD, mm						
Unadjusted	3.45 ± 0.31	3.32 ± 0.28	0.015	3.52 ± 0.30	3.22 ± 0.37	< 0.001
Age-sex adjusted	3.40 ± 0.04	3.36 ± 0.04	0.579	3.51 ± 0.04	3.24 ± 0.07	0.001
LT, mm						
Unadjusted	3.46 ± 0.16	3.50 ± 0.21	0.237	3.34 ± 0.15	3.50 ± 0.16	< 0.001
Age-sex adjusted	3.48 ± 0.03	3.49 ± 0.03	0.824	3.35 ± 0.02	3.48 ± 0.03	0.001
AL, mm						
Unadjusted	24.72 ± 1.14	23.08 ± 0.67	< 0.001	24.66 ± 1.04	23.00 ± 1.04	< 0.001
Age-sex adjusted	24.48 ± 0.13	23.28 ± 0.11	< 0.001	24.53 ± 0.11	23.36 ± 0.19	< 0.001
K, D						
Unadjusted	43.22 ± 1.47	43.02 ± 1.18	0.436	43.09 ± 1.59	43.25 ± 1.42	0.663
Age-sex adjusted	43.14 ± 0.21	43.09 ± 0.19	0.862	43.16 ± 0.18	43.04 ± 0.32	0.761
P, D						
Unadjusted	22.53 ± 1.80	23.88 ± 1.97	< 0.001	21.75 ± 1.65	23.31 ± 2.63	0.014
Age-sex adjusted	22.87 ± 0.29	23.56 ± 0.28	0.110	21.88 ± 0.22	22.92 ± 0.40	0.028

ACD: anterior chamber depth; LT: lens thickness; AL: axial length; K: average keratometry; P: lens power; D: diopters; SD: standard deviation. p-value < 0.05 significant.

Table 3 depicts the relationship of ACD, LT, AL and P with SE, as observed by conducting an age- and sex-adjusted multivariate linear regression analysis. In T1DM patients, eyes with longer AL, larger P, and shallower ACD were associated with a decrease in SE (all p < 0.05), with no significant association identified between LT and myopia (p > 0.05). Meanwhile, the healthy control group consisting of eyes with longer AL and larger P were associated with a decrease in SE (all p < 0.05), while no significant association of ACD and LT with myopia could be found (p > 0.05).

**Table 3:** Multivariate linear regression analysis with Dependent Variable SE adjusted for age and sex in T1DM and Non-DM groups

	R^2^	Independent Factor	Unstandardized	Standardized	95% CI	p
			B	Beta		
T1DM	0.784	ACD	1.275	0.151	0.308 to 2.241	0.010
		LT	0.232	0.017	-1.349 to 1.858	0.777
		AL	-1.719	-0.847	-2.018 to -1.421	< 0.001
		P	-0.347	-0.296	-0.501 to -0.194	< 0.001
Non-DM	0.725	ACD	0.471	0.074	-0.401 to 1.342	0.285
		LT	0.803	0.067	-0.837 to 2.443	0.333
		AL	-1.698	-1.058	-1.996 to -1.400	< 0.001
		P	-0.371	-0.353	-0.561 to -0.181	< 0.001

ACD: anterior chamber depth; LT: lens thickness; AL: axial length; P: lens power; T1DM: type 1 diabetes mellitus; DM: diabetes mellitus; p-value < 0.05 significant

**Fig. 1:**
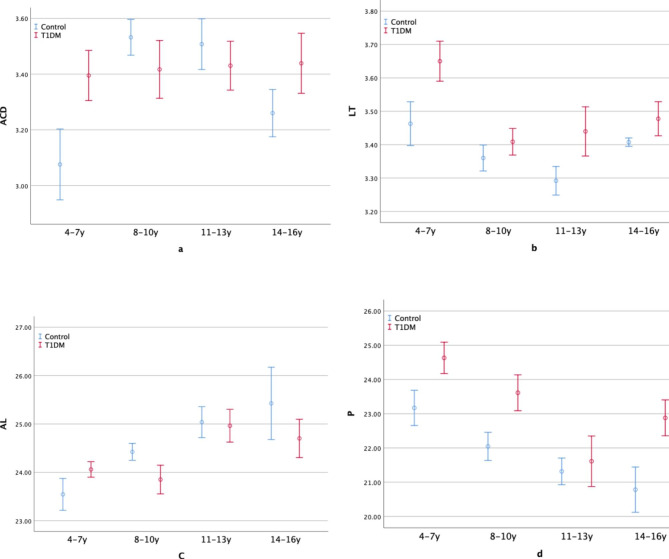
The mean and standard error of ACD (anterior chamber depth, **a)**, LT (lens thickness, **b)**, AL (axial length, **c)**, and P (lens power, **d)** with ages from 4 to 16 years in two myopia subgroups (T1DM: myopia type 1 diabetes mellitus children, Control: myopia controls)

In Fig. 1, the means of the ocular parameters are depicted as undergoing variations with age. The ACD in the control group initially increased with age and then decreased, reaching its peak at around 8–10 years, thus rendering an inverted U-shape. Meanwhile, the T1DM group showed a steadily increasing trend. In contrast, the LT trend for both subgroups displayed a U-shape – the two curves never crossed each other, and LT levels of the T1DM patients were consistently higher than those of the control group. The T1DM group reached its trough at around 8–10 years, while the control group reached its trough at about 11–13 years. Interestingly, the changing trend of AL maintained an approximately linear fashion, with the AL in the control group increasing with age. Notably, AL volatility increased in the T1DM group with age. Moreover, the P curves of the two subgroups were quite different. While it assumed a V-shape in the case of the T1DM group, reaching the bottom at 11–13 years, the P of the control group declined with age, showing a marked reduction over the 4–10 year period, followed by a slow decrease thereafter.

## Discussion

In our previous study [8], we found that an increase in LT is accompanied by a decrease in ACD in T1DM children compared to age- and sex-matched healthy children. Furthermore, no relation was identified between the glycosylated blood haemoglobin (HbA1c) level or DM duration and the ocular parameters (ACD, LT, AL, K and SE). To further analyse the potential myopic impact on developing eyes, four years of recruits with T1DM were included, and a comparison was conducted between the myopia and non-myopia subgroups in the current study. Notably, although the T1DM children were older than the healthy children, age adjustments were made before conducting the comparison between the subgroups, thus ensuring that it does not affect the results.

The lucubrate research revealed that differences in the ocular parameters of T1DM patients and those of a healthy control group exist only in myopic rather than non-myopic subjects. Moreover, in the case of the two myopia subgroups, LT was identified as one of the main differences between myopia T1DM and myopia control children, with the former exhibiting a marked increase in LT compared to the latter. In addition, further analysis of P revealed a marked increase in myopia in T1DM children compared to the myopia control ones. Consequently, a significant question arose: How does the ocular biometry change only for the myopic eye? A comparison of the two healthy control subgroups showed that the myopia control group had a longer AL than the non-myopia control group. The eye seemed to try to refocus on the retina as the flattened LT and smaller P appeared to contend with it. Effectively, the unchanged LT and P made the compensation mechanism more passive in the case of myopia T1DM children, notwithstanding their elongated AL as compared to non-myopia T1DM children. This evidently indicates that myopia may be accelerated in T1DM children. In the Atropine for Treatment of Myopia study [11], atropine-treated eyes displayed less myopic progression, as well as a lesser increase in LT, compared to placebo-treated ones. Furthermore, when atropine was stopped, a marked increase in myopia and an increase in LT were identified in the atropine-treated eyes compared to their counterparts. Although this was suspected to be the pharmacological effect of atropine, the relevance of LT and myopia increases can still be noticed in this context. Our previous study discussed the possible reasons for LT growth in T1DM children to be lens overhydration and its lesser ability to flatten as a result of ciliary muscles [8]. Moreover, MRI tests have proved that the unaccommodated shape of lenses in people with T1DM mimics the accommodated shape of lenses in people without T1DM [12], which is probably why the lens fails to lose power in myopia T1DM children compared to myopia control children.

On adding LT in the linear regression model, no significant relationship was observed between LT and SE in the T1DM and non-DM groups. As for LT’s correlation with P [13], the association between LT and SE was reflected in the impact of P. A larger P was associated with a decrease in SE in both the T1DM and control groups. Furthermore, a significant relationship between ACD and SE was observed in the T1DM groups but not in the non-DM groups. A more relaxed lens, owing to better elasticity, prevented non-DM children from developing myopia. In this context, Gao et al., who examined ocular components before and after cycloplegia, noted a significant decrease in LT and backward movement of the lens after cycloplegia. This means that both an increase in LT and a forward movement of the lens must have occurred during accommodation. At the same time, myopic eyes showed only slight changes in LT and lens movement compared to hyperopic and emmetropic eyes. Moreover, before cycloplegia, myopic eyes exhibited the thinnest LT and the deepest ACD compared to the other groups [14]. In other words, the thinnest LT with the most negligible thickness increase, while the deepest ACD with the most diminutive forward movement in the lens indicates that the myopic eyes maintained the thinnest LT and deepest ACD regardless of accommodation – a result that is consistent with Li et al. [6]. The current study also identified deeper ACD and thinner LT in the myopia control group when compared to the non-myopia control group. However, the ACD and LT of the myopia T1DM children remained unchanged compared to the non-myopia T1DM children. More specifically, the lens in myopia T1DM children could neither move backward nor flatten in relaxed accommodation. Therefore, this study found that P was smaller in the myopia control group compared to the myopia T1DM group (21.61 ± 0.22 vs. 22.72 ± 0.26, p = 0.003), while no significant difference could be identified in the comparison between the myopia T1DM and non-myopia T1DM groups (22.87 ± 0.29 vs. 23.56 ± 0.28, p = 0.110).

Growth trends of the ACD and LT were displayed in the form of a two-phase pattern, with the convex shape in the opposite direction. The trend assumed a U-shape for the LT and an inverted U-shape for the ACD in the case of the myopia control group. Meanwhile, although the growth trend for LT was U-shaped for children with myopia T1DM, it remained constant for ACD. Note that these results are consistent with those of a previous study by SCORM. It was speculated that the first phase of the decrease in LT might have been caused by the stretching of the elongating eyeball, while the increase phase resulted due to thickness growth of the lens, which outpaced the stretching. Furthermore, hyperopic children displayed a flat line as their growth trend, while emmetrope children showed a less concave line than myopic children [15]. In the current study, the myopia control group presented a constantly thinner LT than the myopia T1DM group since the lens in the latter failed to flatten. Furthermore, we inferred that the inverted U-shaped ACD trend could be a result of various reasons. First, the two-phase pattern of the front surface. Second, the first phase of the increase may be caused by the backward movement of the lens in healthy myopes, since the ACD showed an inverted U-shape in children with myopia, while it was constant in children with hyperopia [15] in the SCROM study and in T1DM myopes in the present study. Third, the increase phase may be caused by an increase in AL, but the unidirectional pattern of this increase indicated that it probably had a minor contribution in the case of healthy myopes.

The influence of LT and ACD would eventually impact P. Previous studies have confirmed that P decreases with increased age in children [7, 13, 16, 17]. Our result showed that in myopia controls, P decreased rapidly before ten years and slowed down thereafter. This is consistent with Xiong et al. They also analysed the associations of P with SE or AL in healthy Chinese children to find that it has a positive correlation with SE when SE > -5.00 D, but exhibits a stronger negative association with AL in non-myopes and a weaker negative association in myopes. Their article clarified that before the onset of myopia, P reduces as a compensation for AL elongation to maintain emmetropia, which is a co-effect of changes in the flattening and thinning of the lens and a decline in its internal power due to the compactness of the gradient. However, P loss might be limited, and AL growth might have no endpoint. Myopia develops when the rate of AL growth outpaces the compensatory loss of P. Thus, the compensatory ability of P decreases with age for its natural development and for AL elongating [7]. In the current study, AL increased and P declined with age only in the myopia control group, but it showed a rebound trend after declining until 11–13 years for the myopia T1DM group. In the myopia T1DM group, the lens thickness and gradient refractive index suffer from hyperglycaemia, making the lens less capable of compensating for AL growth, especially after the age of around 11–13 years. As observed in the two myopia subgroups in this study, the same level of SE (p = 0.703), but a smaller P (p = 0.003), was identified in the myopia control group compared to the myopia T1DM group, indicating a more tolerant P in healthy myopes.

Moreover, this study is no exception to the fact that AL elongation contributes to the most SE progression [1]. In the linear regression model, AL elongation played a more dominant role than P in decreasing SE. However, to our knowledge, since the AL change was not caused by T1DM, no further discussion related to this phenomenon has been presented in this study.

Drawing on the significance of the compensatory ability of the lens, the current research found that it remains relatively “inactive” and passive in the process of AL development in T1DM children, making them more likely to develop myopia. However, the current study also has some limitations. First, to eliminate age asymmetry between the T1DM and control groups as a result of the difference in the age groups of the patients from the ophthalmology and endocrinology departments, univariate general linear models and multivariate linear regression analysis were deployed to control for the age effect and ensure that the age difference did not influence the results. However, future studies should consider a larger sample size with age equivalence. Second, while the current study followed a cross-sectional structure, longitudinal follow-ups are required to track the development trends of ocular parameters. Third, the HbA1c level and DM duration were not determined in this study, since their correlation with ocular parameters have already been discussed in a previous study [8], where no statistical significance was found. Hence, no replicate analyses were performed in this study.

To conclude, compared to non-myopia T1DM children, myopia T1DM children failed to achieve deeper ACD and thinner LT, as a result of which the lens could not lose sufficient power to compensate for AL growth. These findings provide evidence that myopia would be accelerated in T1DM children.

## Data Availability

The data used to support the findings of this study are available from the corresponding authors upon request.
